# Supplementary figures and images for: Ideological differences in engagement in public debate on Twitter
Source: PLoS One. 2021 Mar 25;16(3):e0249241. doi: 10.1371/journal.pone.0249241 (PMC7993819; doi:10.1371/journal.pone.0249241)

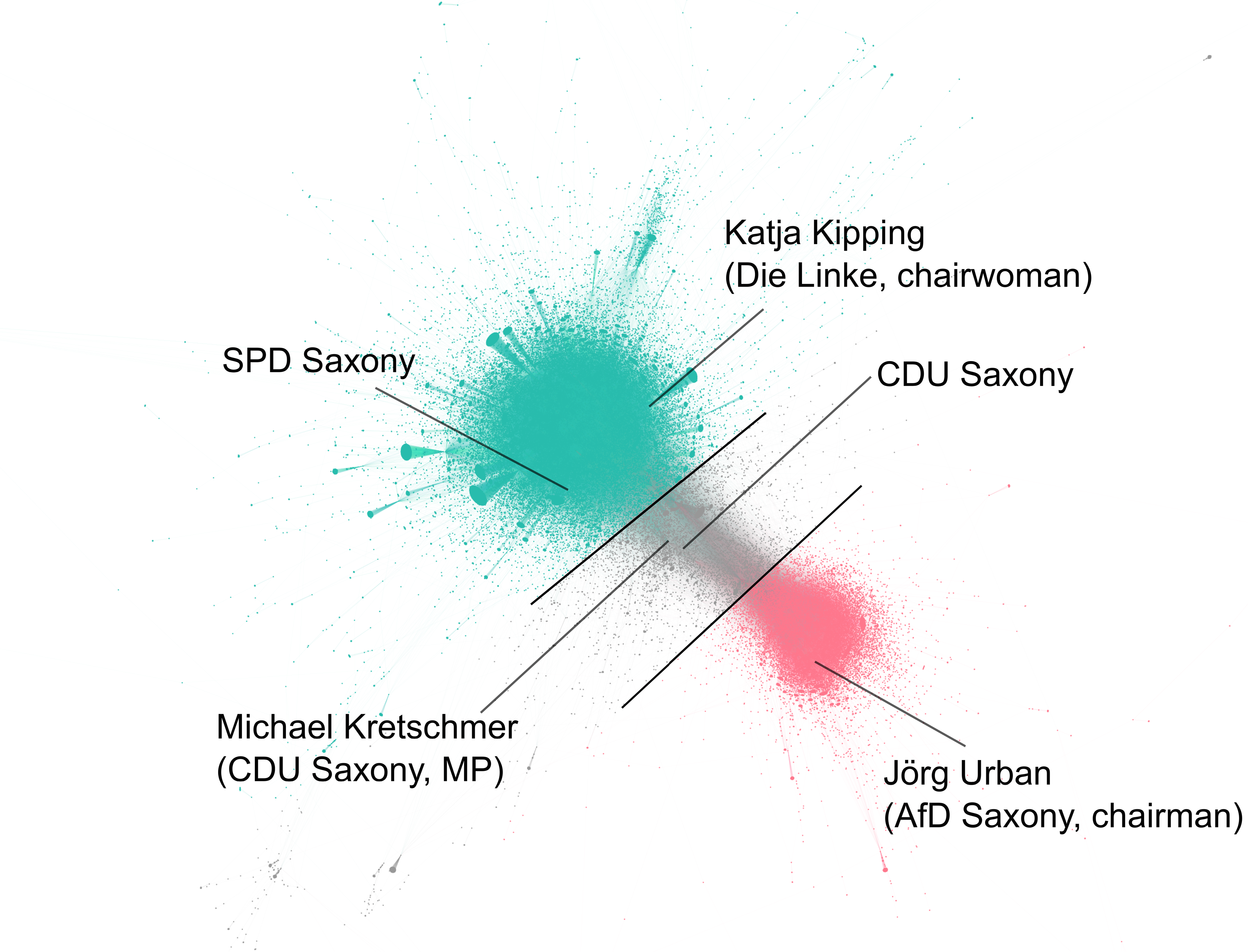

Supplement: S1 Fig — Figure shows the retweet network constructed out of all retweets in the data set between the 1st of July 2019 and then 24th of February 2020. If users were not present in the incident-specific retweet network, it was checked whether this large retweet network contained the users to increase the amount of classified users. Fig 5 exhibits a very similar shape as the incident-specific retweet networks. Out of 88,167 users, 71.8% belonged to the majority cluster, 18.4% to the minority cluster and 9.8% to the intermediate region. (TIF) [file pone.0249241.s001.tif]

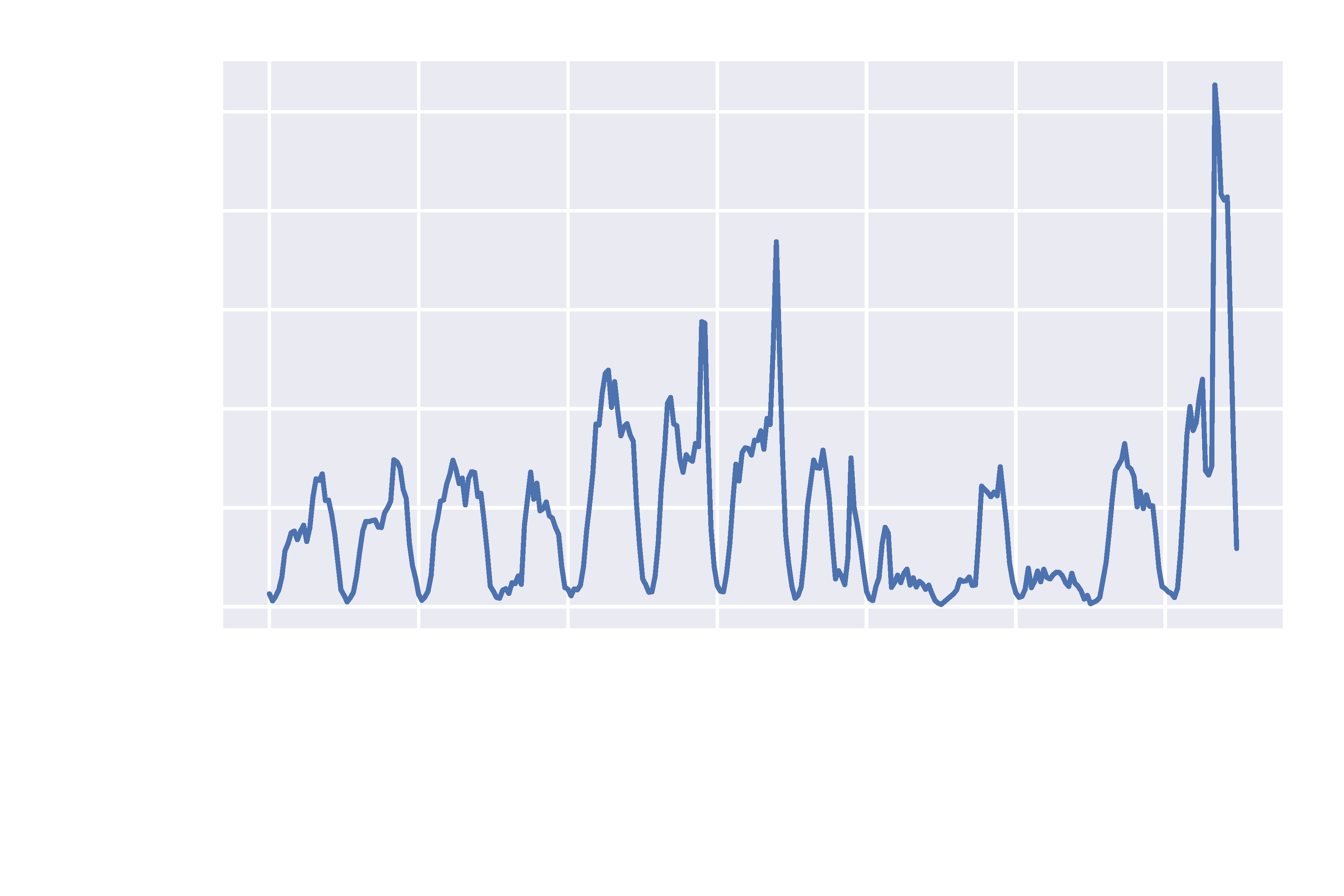

Supplement: S2 Fig — Time series of the collected tweets. As is visible, some tweets were lost due to API problems between the 26th and the 31st of August. (TIF) [file pone.0249241.s002.tif]

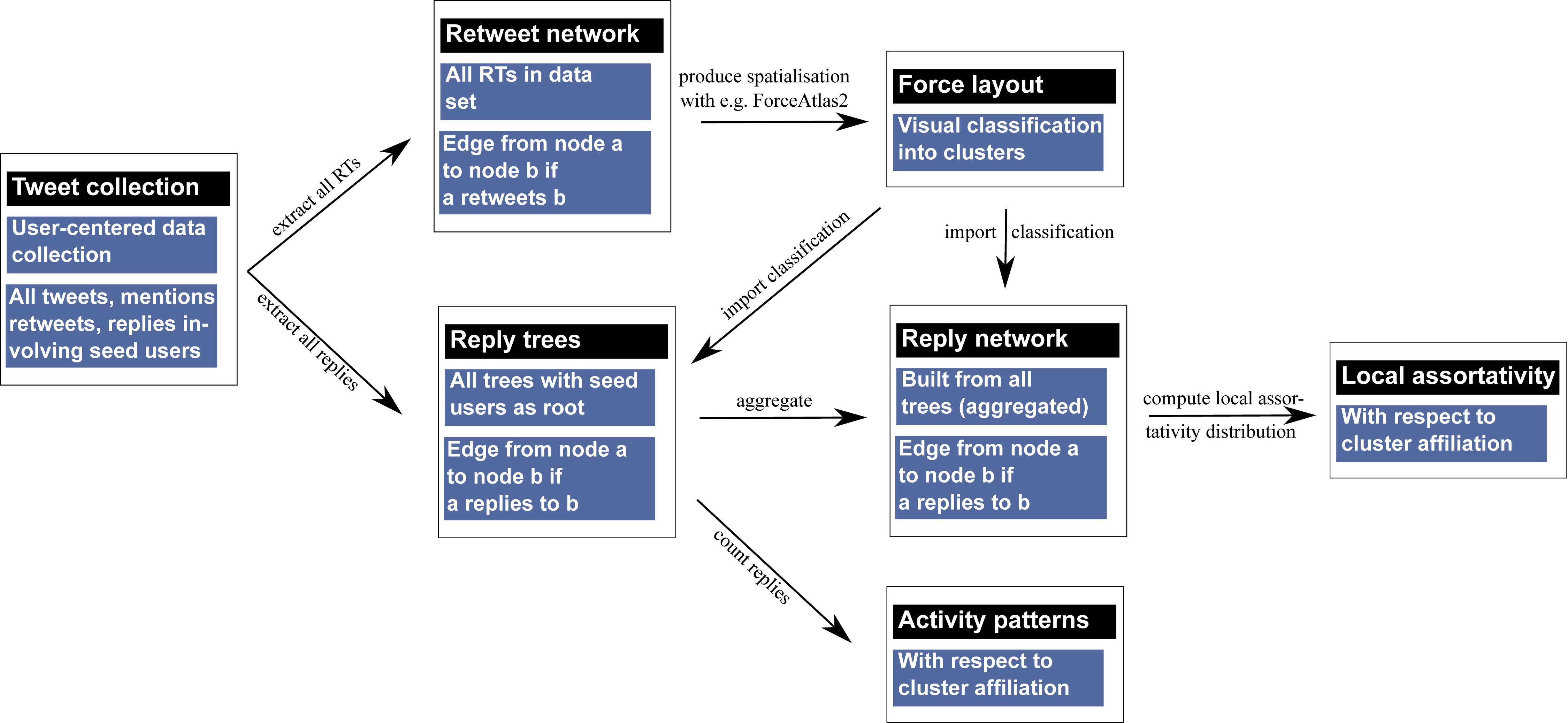

Supplement: S3 Fig — Chart displaying the workflow necessary for the analysis carried out in the paper. (TIF) [file pone.0249241.s003.tif]
