# Supplementary material for: Ideological differences in engagement in public debate on Twitter
Source: PLoS One. 2021 Mar 25;16(3):e0249241. doi: 10.1371/journal.pone.0249241 (PMC7993819; doi:10.1371/journal.pone.0249241)
Supplement: S1 File — Two additional tables with engagement proportions from the large retweet network as well as a retweet network without retweeted replies. (PDF) [file pone.0249241.s004.pdf]

Table 1: Percentage of users in the respective retweet networks (not only incident-specific) that are active in the reply networks (seed users excluded) by cluster.

	Election	NYE
Majority cl.	10.3% (6,737)	3.1% (2010)
Minority cl.	28.1% (4,697)	10.2% (1727)
Intermediate cl.	14.5% (1,431)	4.3% (390)

Table 2: Percentage of users in the respective retweet (networks without replies) that are active in the reply networks (seed users excluded) by cluster.

	Election	NYE
Majority cl.	21.5% (4,040)	16.5% (954)
Minority cl.	44.1% (2,173)	25.6% (386)
Intermediate cl.	24.7% (827)	22.3% (116)

Activity share and possible biases in the data In Table 3 of the main text, we assess whether users from different clusters have different probabilities to get involved in debate. We use the incident-specific retweet network for the statistics there. One can also use the incident-specific retweet cluster combined with the large retweet cluster. The effect then is even more pronounced, as we show in Table 1. We must note here, however, that the number of seed users in the majority pole is, for both data sets, larger than the number of seed users in the minority pole. While we do believe that the snowball-sampling method yielded a representative sample of important figures of Saxon politics and Saxon media outlets on Twitter, if this was not the case, a bias could have been introduced in the activity share of users if many retweets were replies *and* minority pole users mostly commented in reply trees that were initiated by majority pole users. In both data sets, around 20% of the retweets were replies. One can also construct a retweet network without retweets of replies to make sure this bias is eliminated. The effect still holds (Table 2).
